# *Pharmacology Research & Perspectives*: A Decade of Progress

**DOI:** 10.1002/prp2.1057

**Published:** 2023-01-23

**Authors:** Michael F. Jarvis, Emily Davies, Jennifer H. Martin

**Affiliations:** ^1^ Pharmaceutical Sciences University of Illinois at Chicago Chicago Illinois USA; ^2^ Wiley Blackwell Oxford UK; ^3^ The University of Newcastle Hunter Medical Research Institute New Lambton New South Wales Australia

*Pharmacology Research & Perspectives (PR&P)* began publication in 2013 as a gold open‐access journal collaboration between the American Society for Pharmacology and Experimental Therapeutics (ASPET), the British Pharmacology Society (BPS), and Wiley. Since then, the journal has flourished in terms of increased numbers of papers submitted and published, impact factor, and downloaded content. While *PR&P* has always encouraged authors to directly submit their research to the journal, *PR&P* has also welcomed manuscript transfers. This enables authors to transfer submissions to *PR&P* that were originally intended for the established journals of ASPET, BPS, and other supporting biomedical and clinical research journals^1^ but were determined not to be competitive enough by their respective editorial boards. This editorial approach has provided the *PR&P* readership and authors access to rigorous research advances covering both basic and clinical pharmacology in the context of further enabling research reliability and reproducibility, fostering the early clinical development of new chemical entities, and challenging existing pharmacological dogma. *PR&P* has consistently maintained an author‐focused approach to editorial content and the editorial board encourages submissions of original research, short reports, reviews, and topical perspectives that address key issues in pharmacology. Prospective authors are encouraged to carefully review the *PR&P* Author Guidelines when preparing manuscripts for submission, as they are routinely updated with the latest publishing practices and requirements.^2^

In 2019, *PR&P* received its first Impact Factor (2.05), and this metric has steadily increased to 2.97 for 2022. Using 2019 as a benchmark, submissions to *PR&P* have increased between 200–300% in the succeeding two years. The number of papers published have also significantly increased (50–100%) over this time. Consequently, the acceptance rate for publication in *PR&P* has decreased to approximately 30% of submitted papers as the editorial board has continued its work to further build the scientific quality of the journal. Authors should be aware that the acceptance rate for transferred papers from supporting journals is typically higher. In all cases, transferred papers accepted by *PR&P* communicate rigorous science that is well within the scope of PR&P's strategic goals and authors have effectively resolved the original peer review feedback they have received. In response to the continued growth of the journal, *PR&P* has also recently established a group of Senior Editors representing the diversity of pharmacology interests across the globe to oversee peer‐review and ensure authors continue to receive timely and constructive critiques of their manuscripts.

Improving the author's experience is central to the *PR&P* strategy. Over the past few years the journal has implemented new workflows and processes to reduce the post‐acceptance time to publication to an average of 23 days for 2022. While the time to first decision is already very competitive, at an average of just four days for 2022, *PR&P* will continue to prioritize decreasing the time to final decision with the help of the newly established Senior Editors. The journal also aims to reduce time spent by authors on the initial submission process, by introducing a new author facing submission platform, Research Exchange^3^ which has already been piloted and rolled out across over 400 Wiley journals. To further reduce author time spent resubmitting manuscripts, and to reduce reviewer demand, *PR&P* will continue to support transfer networks between journals, inclusive of expanding this network to offer referral options to papers deemed out of scope for *PR&P*.

Since its inception, *PR&P* has actively encouraged submission of papers that address all aspects of drug action including new hypotheses, mechanisms of action, target identification, target validation, lead optimization, and clinical development including translational medicine, quality use of medicines projects, drug repurposing studies, and pharmacovigilance. Another major focus has also been on the enhancement of research reliability through the publication of replication studies and negative results from well‐designed experimental research projects. Over the last several years, *PR&P* has provided readers with multiple dedicated issues that cover these and other cutting‐edge pharmacological research topics (Table 1). Of particular importance to the strategic goals of *PR&P* is highlighting research contributions from different geographical regions and providing a venue for early career researchers to submit original research and topical reviews that are based on their graduate and/or post‐doctoral experiences. *PR&P* has also partnered with other BPS journals to generate special virtual issues in the areas of sex as an independent variable and COVID‐19 research. More recently, *PR&P* has championed the publication of advances in pharmacology education including new instructional models, curricula, and best practices. Readers are encouraged to access the recently published BPS statement on Inclusive Pharmacology Education.^4^

**TABLE 1:** Selected Themed/Special Issues of *Pharmacology Research & Perspectives*.

**Re‐purposed therapies in COVID‐19: The overlap of biology, physiology, pharmacology and clinical pharmacology** March 2022 **Glia Pharmacology in Asia & Beyond** November 2021 **Research Highlights from South America** October 2021 **Early Career Researcher** January 2020 **Neurodegeneration & Neuroprotection** December 2020 **Highlights in “Negative Findings”** October 2020 **Highlights in Pharmacological Research Methodology** July 2020 **Perspectives and Reviews** March 2020

Going forward, *PR&P* will continue to build upon the strategic initiatives noted above. Readers can expect new thematic issues highlighting pharmacology research from the African Continent, a special issue on Pharmacology Education and Innovation, and further efforts to advance research generated by early career scientists. To assist potential authors in considering new submissions to *PR&P*, please see the thematic tips outlined in Table 2. Prospective authors are also encouraged to contact *PR&P* regarding special projects such as key scientific meeting summaries, risk/benefit profiles for new drug classes, or new technological advances that advance understanding of drug mechanisms.

**TABLE 2:** PR&P Thematic Priorities for Potential Authors.

*PR&P* encourages submissions covering the general topics below Drug mechanism of action Comparative pharmacology e.g. species, receptor subtypes, expression systems All areas of nonclinical and clinical pharmacology: including first‐in‐human and proof‐of‐concept (positive or negative outcome) studies, regulatory authority required studies Drug discovery and enabling science: including new target identification and validation (including positive and negative outcomes), lead optimization, drug repurposing, and data that advances or terminates drug discovery projects Novel pharmacological hypotheses Frontiers in translational medicine: articles that enable or review accumulating preclinical data on emerging clinical candidates Replication studies: research that supports or refutes key findings (failed replication) Papers that are out of scope for *PR&P* Research on natural product‐derived reagents that do not involve quantitatively isolated and characterized compounds In‐silico or bioinformatic analyses that do not report supporting biological data Biochemical signaling research that lacks pharmacological interventions Clinical case reports or studies that do not involve a pharmacological treatment Meta‐analyses of nonclinical or clinical research without a compelling mechanistic hypothesis
